# Sex similarities and divergences in systemic and muscle iron metabolism adaptations to extreme physical inactivity in rats

**DOI:** 10.1002/jcsm.13547

**Published:** 2024-07-24

**Authors:** Mathieu Horeau, Melissa Delalande, Martine Ropert, Patricia Leroyer, Brice Martin, Luz Orfila, Olivier Loréal, Frédéric Derbré

**Affiliations:** ^1^ Laboratory “Movement Sport and Health Sciences” EA7470 University of Rennes/ENS Rennes France; ^2^ INSERM, University of Rennes, INRAE, UMR 1317 Nutrition Metabolisms and Cancer (NuMeCan) Institute Rennes France; ^3^ Elemental Analysis and Metabolism of Metals (AEM2) Platform Univ Rennes CHU Pontchaillou Rennes France

**Keywords:** Disuse, Haemoglobin, Metals, Spaceflight, Trace elements

## Abstract

**Background:**

Previous data in humans suggest that extreme physical inactivity (EPI) affects iron metabolism differently between sexes. Our objective was to deepen the underlying mechanisms by studying rats of both sexes exposed to hindlimb unloading (HU), the reference experimental model mimicking EPI.

**Methods:**

Eight‐week‐old male and female Wistar rats were assigned to control (CTL) or hindlimb unloading (HU) conditions (*n* = 12/group). After 7 days of HU, serum, liver, spleen, and soleus muscle were removed. Iron parameters were measured in serum samples, and ICP‐MS was used to quantify iron in tissues. Iron metabolism genes and proteins were analysed by RT‐qPCR and Western blot.

**Results:**

Compared with control males, control females exhibited higher iron concentrations in serum (+43.3%, *p* < 0.001), liver (LIC; +198%, *P* < 0.001), spleen (SIC; +76.1%, *P* < 0.001), and transferrin saturation (TS) in serum (+53.3%, *P* < 0.001), contrasting with previous observations in humans. HU rat males, but not females, exhibited an increase of LIC (+54% *P* < 0.001) and SIC (+30.1%, *P* = 0.023), along with a rise of H‐ferritin protein levels (+60.9% and +134%, respectively, in liver and spleen; *P* < 0.05) and a decrease of TFRC protein levels (−36%; −50%, respectively, *P* < 0.05). HU males also exhibited an increase of splenic *HO‐1* and *NRF2* mRNA levels, (*p* < 0.001), as well as HU females (*P* < 0.001). Concomitantly to muscle atrophy observed in HU animals, the iron concentration increased in soleus in females (+26.7, *P* = 0.004) while only a trend is observed in males (+17.5%, *P* = 0.088). In addition, the H‐ferritin and myoglobin protein levels in soleus were increased in males (+748%, *P* < 0.001, +22%, *P* = 0.011, respectively) and in females (+369%, *P* < 0.001, +21.9%, *P* = 0.007, respectively), whereas TFRC and ferroportin (FPN) protein levels were reduced in males (−68.9%, *P* < 0.001, −76.8%, *P* < 0.001, respectively) and females (−75.9%, *P* < 0.001, −62.9%, *P* < 0.001, respectively). Interestingly, in both sexes, heme exporter FLVCR1 mRNA increased in soleus, while protein levels decreased (−39.9% for males *P* = 0.010 and −49.1% for females *P* < 0.001).

**Conclusions:**

Taken together, these data support that, in rats (1) extreme physical inactivity differently impacts the distribution of iron in both sexes, (2) splenic erythrophagocytosis could play a role in this iron misdistribution. The higher iron concentrations in atrophied soleus from both sexes are associated with a decoupling between the increase in iron storage proteins (i.e., ferritin and myoglobin) and the decrease in levels of iron export proteins (i.e., FPN and FLVCR1), thus supporting an iron sequestration in skeletal muscle under extreme physical inactivity.

## Introduction

Physical deconditioning occurs in bedridden patients and astronauts in a space environment, particularly as a result of prolonged exposure to extreme physical inactivity. This deconditioning significantly affects the hospitalization duration of bedridden patients, particularly in older adults,^1^ and also impacts astronauts' ability to perform their missions during extravehicular activities. In both cases, prolonged bedrest and microgravity promote an early‐onset anaemia and skeletal muscle atrophy.^2^, ^3^, ^4^, ^5^ Interestingly, haemoglobin in red blood cells (comprising 60–70% of body iron) and myoglobin in muscle fibres (15–20%), essential for oxygen transport, delivery and storage, collectively account for the majority of iron content in the body.^6^

The objective of the mechanisms of both systemic and cellular iron metabolism regulation is to ensure sufficient delivery of iron to cells, and also to avoid iron excess that exposes to toxicity through the production of reactive oxygen species (ROS) and related oxidative damage.^7^, ^8^ At systemic level, hepcidin, a peptide secreted in plasma, mainly by the liver,^9^ plays a critical role in the systemic regulation of iron metabolism. It promotes the internalization of the cell surface iron exporter ferroportin (FPN), thus limiting iron absorption by enterocytes in the duodenum and promoting cellular iron sequestration mainly in liver and spleen macrophages (Nemeth 2004), the enterocytes and macrophages being the main providers of iron in plasma. Thus, hepcidin controls the level of saturation of transferrin, the plasma protein involved in iron delivery to cells. At cellular level, iron metabolism is primarily regulated by the IRE/IRP system,^10^ which controls the expression of genes containing iron‐responsive elements (IREs) (1) at mRNA level such as transferrin receptor protein‐1 (TRFC) and divalent metal transporter 1 (DMT1) that play a role in cell iron uptake, or (2) at protein level such as ferritin and FPN, that play a role in cell iron storage and egress, respectively. Furthermore, the feline leukaemia virus subgroup C receptor 1 gene (*Flvcr1*) has been reported to encode the FLVCR1 protein, a cell membrane heme exporter crucial for protecting erythrocyte precursors against potential heme accumulation that may lead to toxicity.^11^ However, the potential role of *Flvcr1* gene in other cell types, including skeletal muscle fibres, remains unclear.

Recent studies show that extreme physical inactivity rapidly affects the iron distribution in young men and male rats.^4^, ^12^, ^13^, ^14^ After only few days, increases in plasma iron availability (i.e., transferrin saturation), as well as in spleen iron concentrations (SIC), and serum hepcidin levels are thus reported in male rats exposed to hindlimb unloading, as well as in young men exposed to bedrest or dry immersion.^12^, ^13^, ^14^, ^15^ These iron metabolism disturbances appears persisting over several weeks and months, and are progressively associated to an abnormal iron accumulation in the liver and bones in rats.^2^, ^12^, ^16^, ^17^ Whereas iron metabolism systemic parameters are modulated during extreme physical inactivity, the underlying mechanisms remain poorly understood.

Given the known differences in iron metabolism between males and females,^18^, ^19^ it is crucial to explore how both sexes adapt to extreme physical inactivity and to characterize the underlying mechanisms. Recent data from the pilot randomized clinical trial AGBRESA, involving a small panel of males and females exposed to 2 months of bedrest, suggest differences in systemic iron metabolism control, particularly regarding hepcidin expression levels.^12^

Therefore, our objective was to investigate the impact of extreme physical inactivity on the regulation of liver, splenic, and muscle iron metabolism, particularly in relation to skeletal muscle atrophy. We also paid attention to the sex similarities and divergences between males and females. For this purpose, we explored male and female rats exposed to 7 days of hindlimb unloading (HU), the reference model to mimic the effects of extreme physical inactivity in rodents.

## Methods

### Study approval

The animal experiments received approval from the Rennes committee on Ethics in Research (authorization 30783‐2021033022287826) in accordance with the European directives (86/609/European Economic Community).

### Animals and experimental procedures

Young male (*n* = 24) and female (*n* = 24) Wistar rats (8‐week‐old, Janvier Labs, Le Genest St Isle, France) were housed in a temperature‐controlled room (21 ± 2°C) with a 12‐h:12‐h light dark cycle. The animals were provided with standard rodent chow and water ad libitum. After a week of acclimatization, the animals were randomly assigned to four groups: control females (CTL‐F, *n* = 12), control males (CTL‐M, *n* = 12), hindlimb unloaded females (HU‐F, *n* = 12) and hindlimb unloaded males (HU‐M, *n* = 12). Hindlimb unloading was achieved using Morey's tail‐suspension model.^20^ After 7 days of exposure, the rats were deeply anaesthetized with a ketamine‐xylazine‐butorphanol cocktail. The liver, spleen and soleus were dissected, weighed and then either frozen in liquid nitrogen or fixed in 4% paraformaldehyde (PFA). Intracardiac blood was collected into dry tubes. Haemoglobin concentration was determined using HemoCue® 201 + system (HemoCue, AB, Meaux, France). Three microhematocrit tubes were filled from blood samples for haematocrit (Hct) determination by centrifugation (3000 RPM, 10 min, RT). The blood was then centrifuged (1500 *g*, 10 min, 4°C) for serum sampling.

### Blood and serum analyses

Serum iron and unsaturated iron‐binding capacity (UIBC) were measured in the biochemistry laboratory of Rennes Pontchaillou Hospital using the Cobas 8000 analyser Roche® (Cobas® reagents 03183696 122 and 04536355 190, respectively, Roche Diagnostics, Meylan, France). Serum transferrin saturation was calculated as [serum iron/(serum iron + UIBC)] × 100. Liver, spleen, and soleus iron concentrations were quantified by ICP‐MS as previously reported.^21^ Serum hepcidin (S‐1483, BMA Biomedicals, Switzerland) concentrations were measured by enzyme‐linked immunosorbent assay, according to the manufacturer's instructions.

### Iron quantifications in liver, spleen, and skeletal muscles

Iron concentrations in the liver, spleen, and soleus were quantified at the AEM2 Platform (Université de Rennes, CHU Rennes) by inductively coupled plasma mass spectrometry on an X‐Series IO from ThermoFisher Scientific equipped with collision cell technology (AEM2 platform UF3142), as previously described.^21^

### Cytosolic protein extraction and Western blotting

Liver, spleen, and soleus were ground in liquid nitrogen, and the resulting powder was used to perform protein or RNA extraction. Cytosolic protein extraction was performed as previously reported.^21^ Samples containing 50–100 μg of proteins were resolved on 8 to 14% SDS‐PAGE (depending on the protein molecular weight). After protein transfer on nitrocellulose membrane, overnight incubation was performed at 4°C with the appropriate primary antibodies (Table 1). All blots were scanned using the Odyssey Imaging System (LI‐COR), and densitometric analysis of the bands was conducted using ImageStudioLite software version 5.2.5 (LI‐COR Biosciences). All blots were corrected for loading based on HSC70 expression.

**Table 1:** List of primary antibodies used for western blotting

Antibodies	Reference	Source	Dilution	Molecular weight (kDa)	
H‐ferritin	Abcam	65080	Rabbit	1:1000	21
HSC70	Santa Cruz	Sc‐7298	Mouse	1:5000	70
FPN	Novus	NBP1‐21502	Rabbit	1:1000	62
FPN	Alomone	261‐275	Rabbit	1:1000	62
Myoglobin	Abcam	Ab77232	Rabbit	1:1000	17
FLVCR1	Invitrogen	PA590258	Rabbit	1: 1000	60
Cytochrome c	Santa cCruz	Sc‐13156	Rabbit	1: 1000	15
TFRC	Invitrogen	RRID:AB_2533029	Mouse	1:1000	90
Anti‐mouse IgG	Cell Signaling	#5257	Mouse	1:20 000	‐
Anti‐rabbit IgG	Cell Signaling	#5151	Rabbit	1:20 000	‐

### RNA extraction and reverse transcription–real‐time PCR

Quantitative PCR (qPCR). qPCR was conducted as previously described.^21^ Briefly, total RNA extractions from liver, spleen, and soleus were performed with Trizol®, according to the manufacturer's instructions (Invitrogen, Vilvoorde, Belgium). Samples were analysed in duplicate in a 10 μL reaction volume containing 4.8 μL IQSybrGreen SuperMix (Bio‐Rad), 0.1 μL of each primer (100 nM final) and 5 μL of cDNA. Primer sequences are reported in Table 2. Target genes were normalized using three reference genes according to geNorm analysis^22^ and expressed relative to the control group. The references genes were hypoxanthine phosphoribosyltransferase 1 (Hprt1), TATA‐binding protein (*Tbp*), and Glucuronidase Beta (*Gusb*).

**Table 2:** Primer design for qPCR

Gene	Reference	Forward primer	Reverse primer
*Tfrc*	NM_022712.1	GAGGAGGTGCTTCAGAGTGCT	CGGGTGTATGACAATGGC
*Fpn*	NM_133315.2	TTGCTGCTAGAATCGGTCTTTG	TCGAGAAGGTAGTTCATGGAGTTC
*Flvcr1*	XM_039091503	GTTCCAAGCTGATTGCCCA	TTCTACCAAGTGTCGCCGC
*Nrf2*	NM_001399173.1	CCCGAGTTACAGTGTCTTAATACG	TGGCTGGCATCATCCGT
*Ho‐1*	NM_012580.2	GGCTTTAAGCTGGTGATGGC	CAGGTAGCGGGTATATGCGTG
*Myoglobin*	NM_021588.2	ATCAGTCTATTTAAGGCTCACCC	GGATCTCAGCAGCATGTTGTC
*Hprt1*	NM_012583.2	CTGATTATGGACAGGACTGAAAGAC	CCAGCAGGTCAGCAAAGAACT
*Gusb*	NM_017015.3	TCGAACAATCGGTTGCAGG	AGCCAATGAAGTTCCGAAGC
*Tbp*	NM_001004198.1	GGGATTGTACCACAGCTCCA	CAGCAAACCGCTTGGGATTA
*Rpl4*	NM_022510.1	CGAGCACCACGCAAGAAGAT	ATTCCTAGCCTGGCGGAGAA

### Histology and immunohistochemistry staining

To visualize iron deposits, deparaffinized spleen and liver sections were stained with Perls' Prussian Blue. To visualize fibre cross sectional area (CSA), muscle cuts were performed in the wider part of the soleus muscle. Subsequently, these sections were stained using the wheat germ agglutinin (WGA) method for reticulin stain. Fibre CSA was determined from at least 1000 fibres per muscle.

### Statistical analysis

All data are presented as individual values and median. The normality of data distribution and homoscedasticity were assessed using the Shapiro–Wilk test and Bartlett's test, respectively. To determine the effects of hindlimb unloading (HU), sex and interaction, each experimental group was compared using a two‐way ANOVA (general linear model) followed by the Tukey's multiple comparison test with adjusted *P*‐value. If the assumptions of normality and/or homoscedasticity were violated, a Box‐Cox transformation was applied. Data were analysed using RStudio (version 1.4.1106) and graphs were created using Prism 8 (Version 8.2.1).

## Results

**Figure 1:**
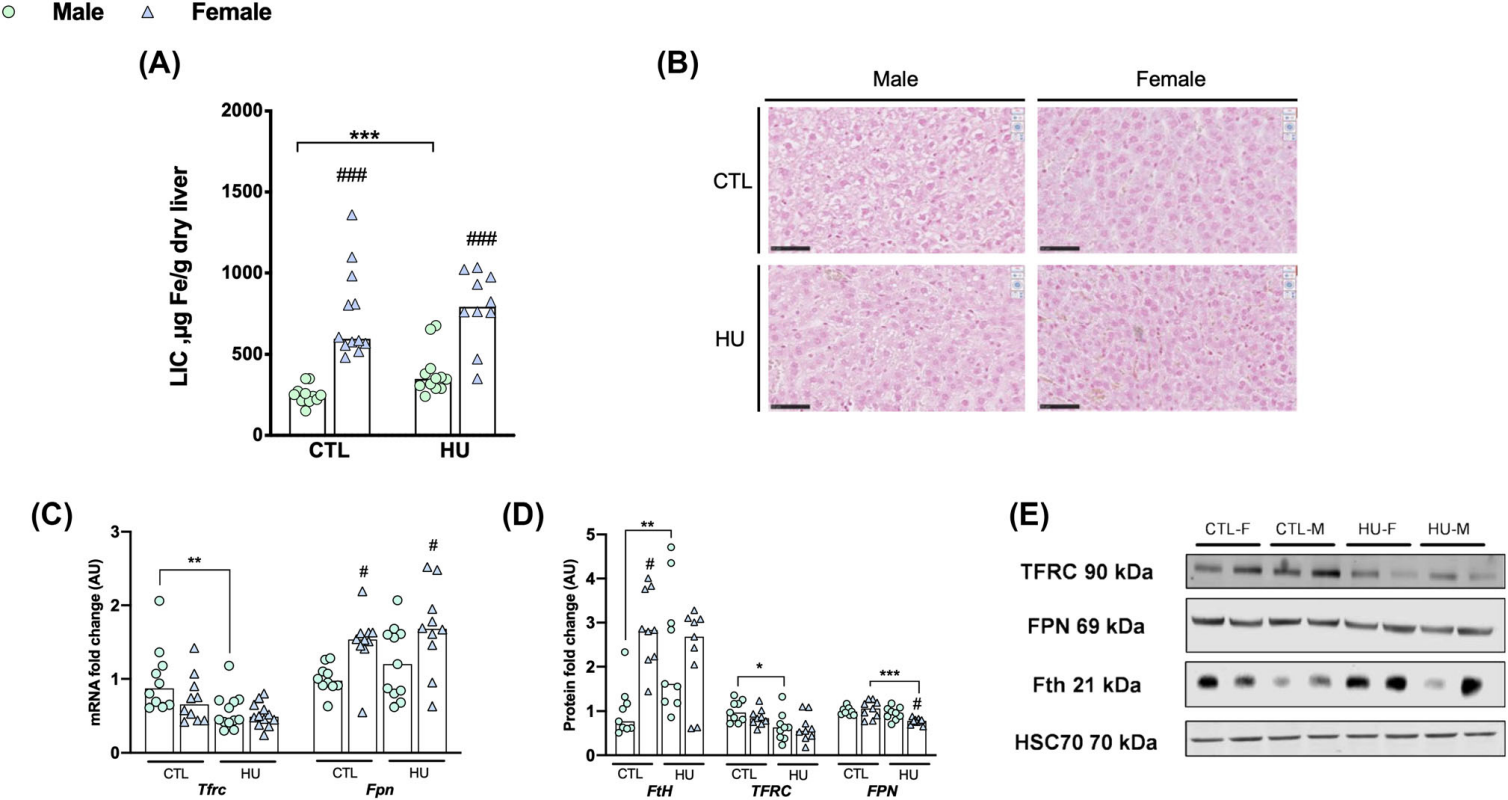
Liver iron concentration is increased only in males exposed to HU. Data were represented as individual value and median. Significance was checked using two‐way ANOVA test and Tukey's post‐hoc test. Global effects of ANOVA are represented on top of each graph. Differences between males and females in each condition (Tukey's post‐hoc tests): ^#^ *P* < 0.05; ^##^ *P* < 0.01; ^###^ *P* < 0.001. Differences between control groups and HU groups (Tukey's post‐hoc tests): *P < 0.05; ^**^ *P* < 0.01; ^***^ *P* < 0.001 (*n* = 12 male and *n* = 12 female). (A) Liver iron concentrations. (B) Reference image of liver histological blue Perls staining. (C) Expression levels of *Trfc* and *Fpn* mRNA. (D) Expression levels of FtH, TFRC and FPN proteins. (E) Liver Western blot reference images. FPN, ferroportin; FtH, ferritin H; LIC, liver iron concentration; Tfrc, transferrin receptor C.

**Figure 2:**
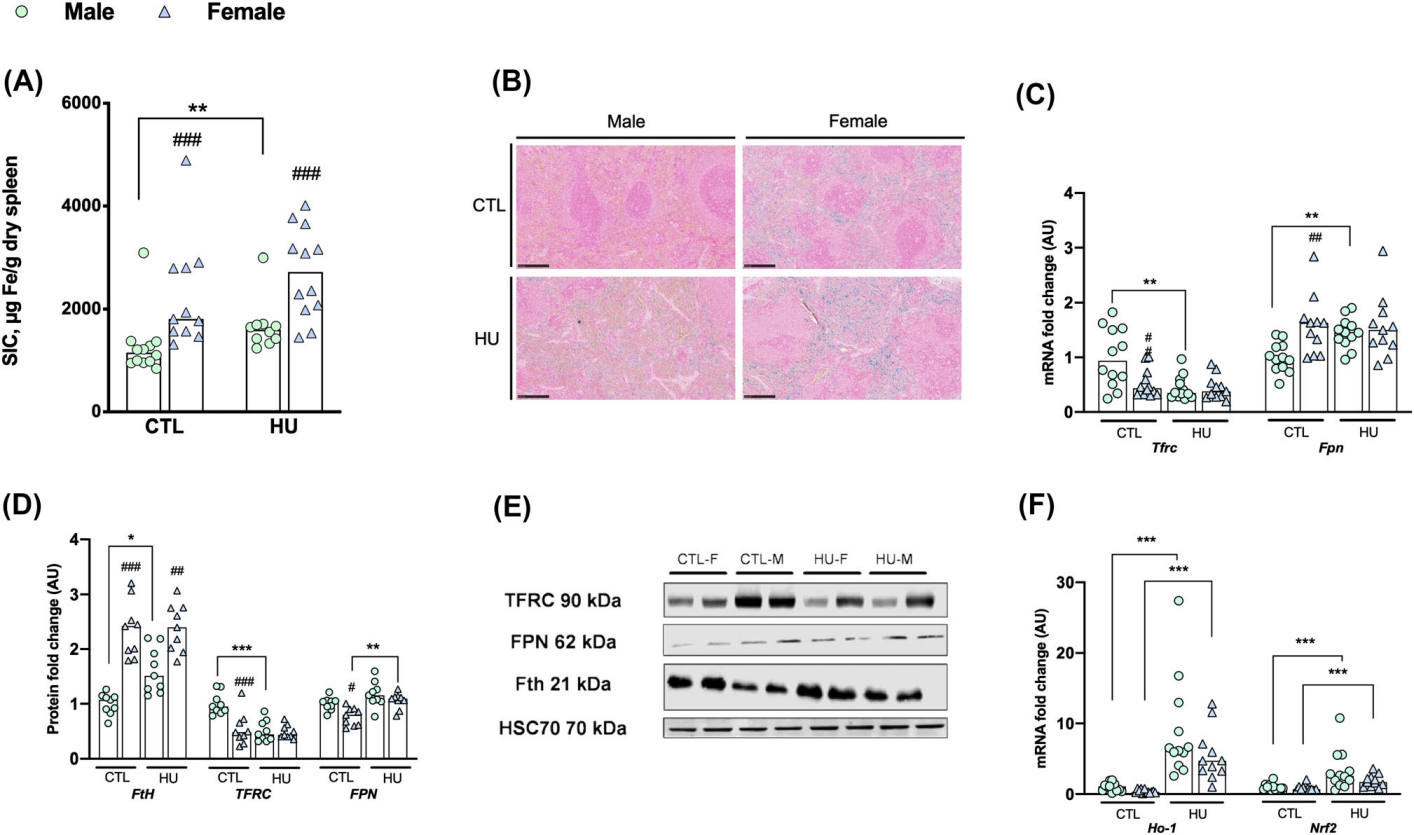
Erythrophagocytosis seems increase the splenic iron concentration specifically in HU males. Data were represented as individual value and median. Significance was checked using two‐way ANOVA test and Tukey's post‐hoc test. Global effects of ANOVA are represented on top of each graph. Differences between males and females in each condition (Tukey's post‐hoc tests): #*P* < 0.05; ##*P* < 0.01; ###*P* < 0.001. Differences between control group and HU group (Tukey's post‐hoc tests): *P < 0.05; **P < 0.01; ***P < 0.001 (*n* = 12 male and *n* = 12 female). (A) Spleen iron concentrations. (B) Reference image of spleen histological blue Perls coloration. (C) Expression levels of *Trfc* and *Fpn* mRNA. (D) Expression levels of FtH, TFRC and FPN proteins. (E) Spleen Western blot reference images. (F) Erythrophagocytosis indirect markers mRNA expression. FPN, ferroportin; ho‐1, heme‐oxygenase 1; NRf2, nuclear factor erythroid 2 like 2; SIC, spleen iron concentration; Tfrc, transferrin receptor C.

**Figure 3:**
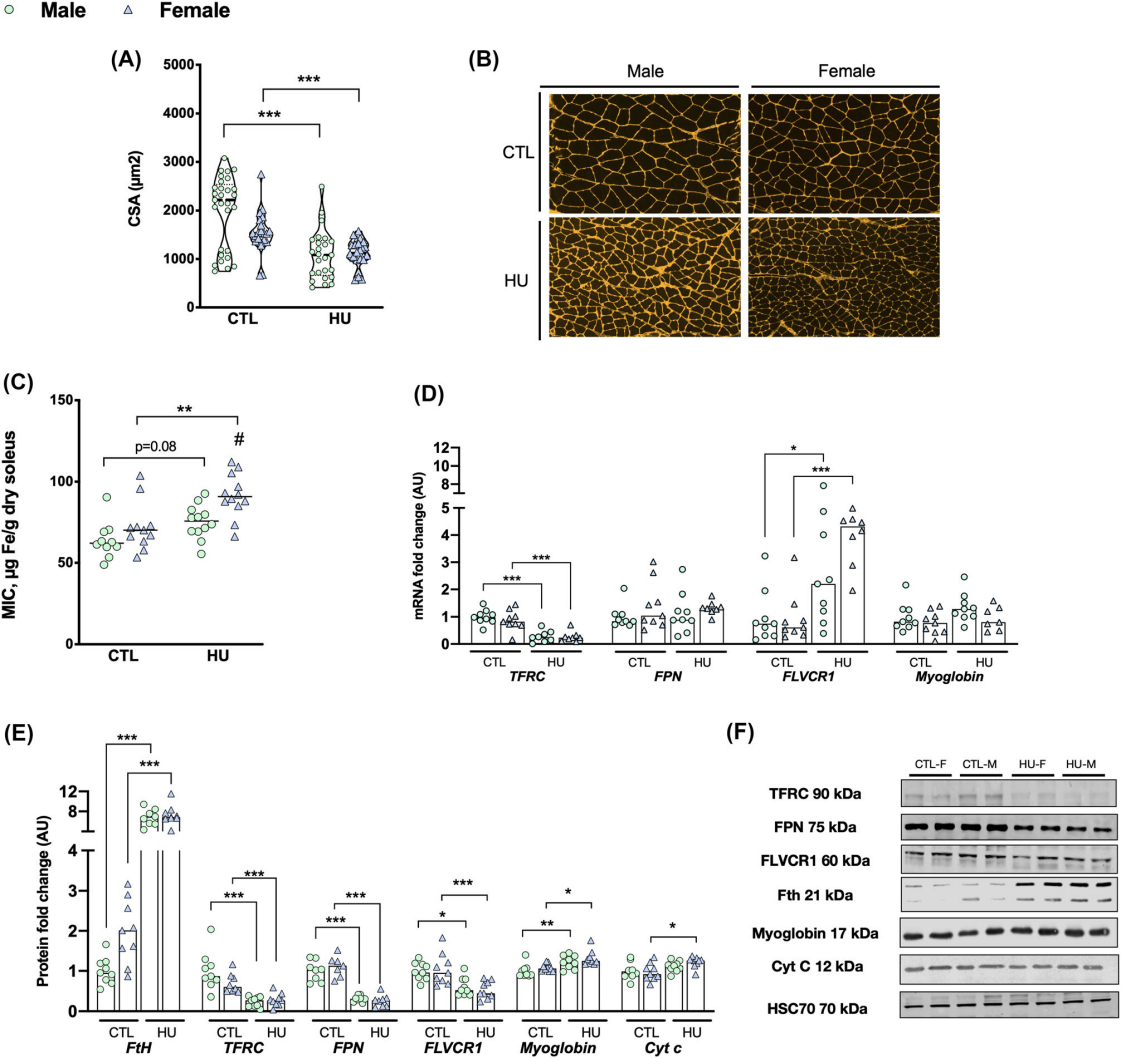
Muscle atrophy due to extreme physical inactivity induces soleus iron sequestration in both sexes. Data were represented as individual values and median. Significance was checked using two‐way ANOVA test and Tukey's post‐hoc test. Global effects of ANOVA are represented on top of each graph. Differences between males and females in each condition (Tukey's post‐hoc tests): ^#^ *P* < 0.05. Differences between control group and HU group (Tukey's post‐hoc tests): *P < 0.05; ^**^ *P* < 0.01; ***P < 0.001 (*n* = 12 male and *n* = 12 female). (A) Soleus cross sectional area (μm^2^). (B) Reference image of histological WGA immunostaining. (C) Soleus iron concentrations. (D) Expression levels of *Trfc*, *Fpn*, *Flvcr1*, and *myoglobin* mRNA. (E) Expression levels of FtH, TFRC, FPN, FLVCR1, myoglobin, and cytochrome c proteins. (F) Soleus Western blot reference images. cyt c: cytochrome c; FLVCR1, feline leukaemia virus subgroup C cellular receptor 1; FPN, ferroportin; MIC, muscle iron concentration; Tfrc: transferrin receptor C.

To compare iron metabolism between males and females exposed to extreme physical inactivity, we explored iron parameters (Table 3), in serum, and also in liver and spleen, the main iron storage organs (Figure 1). We also investigated the mechanisms involved in iron accumulation in spleen (Figure 2). Finally, we compared between sexes the adaptations occurring in soleus, an iron‐rich oxidative skeletal muscle (Figure 3).

**Table 3:** Blood red cells and serum iron parameters

	CTL		HU		2way ANOVA		
	Male	Female	Male	Female	HU	Sex	HU*Sex
Iron (μmol/L)	37.31 ± 7.0	53.45 ± 11.3^###^	32.71 ± 7.0	50.13 ± 11.7^###^	NS	*P* < 0.001	NS
UIBC (μmol/L)	44.02 ± 8.6	23.55 ± 8.4^###^	38.6 ± 8.8	28.43 ± 11.4^##^	NS	*P* < 0.001	NS
TS (%)	46.02 ± 8.4	69.19 ± 10.5^###^	46.10 ± 10.9	63.86 ± 14.1^##^	NS	*P* < 0.001	NS
Hepcidin (pg/ml)	1.32 ± 0.2	1.73 ± 0.2^###^	1.62 ± 0.3*	1.82 ± 0.4	0.019	*P* < 0.001	0.039
Haemoglobin	14.63 ± 1.6	14.15 ± 1.3	16.65 ± 1.8	14.33 ± 2.0	NS	NS	NS
Hct (%)	45.12 ± 4.2	43.39 ± 2.9	49.58 ± 3.9*	43.61 ± 6.4	0.002	0.013	NS

The results are expressed as the mean ± SD, *n* = 12/group. Differences between males and females in each condition (Tukey's post‐hoc tests). Differences between control groups and HU groups (Tukey's post‐hoc tests).

Hct, haematocrit; TS, transferrin saturation; UIBC, unsaturated iron‐binding capacity.

* *P* < 0.05,

^##^ *P* < 0.01, and

^###^ *P* < 0.001.

### Blood iron parameters during extreme physical inactivity in female and male rats

Blood iron parameters in control and HU male and female rats are presented in Table 3.

In control animals, we found that serum iron levels and transferrin saturation are higher in females compared with males (+43.3%, *P* < 0.001 and +53.3%, sex: *P* < 0.001, respectively). We also observed higher serum hepcidin levels in control females compared with control males (+31.3%, *P* < 0.001).

Seven days of HU do not significantly affect the serum iron and transferrin saturation levels in both females and males rats compared with their respective controls. The serum hepcidin levels increase in HU males compared with control males (+23.1%, Sex*HU: *P* = 0.039), whereas they remain unchanged between the two female groups.

### Extreme physical inactivity differently affects hepatic iron metabolism in female and male rats

The liver being one of the organs that stores excess iron, we explored the impact of sex and extreme physical inactivity on the expression of iron metabolism genes and proteins in the liver (Figure 1).

In the liver of control animals, we found that liver iron concentration (LIC) was higher in females than in males (+198%, sex: *P* < 0.001, Figure 1A,B), associated with higher levels of H‐ferritin protein (+188%, sex: *P* = 0.003, Figure 1D,E). Whereas FPN mRNA levels are higher in control females (+52%, sex: *P* < 0.001, Figure 1C), the protein levels of FPN do not differ between control females and males (Figure 1D,E).

Exposure to HU induced different responses between both sexes. In males, contrary to females, LIC increase significantly (+54% vs. +5.8%, Sex*HU: *P* = 0.025, Figure 1A,B) and H‐ferritin protein levels also increase (+138% vs. −24%, Sex*HU: *P* = 0.008, Figure 1D,E). Concomitantly, in both sexes, HU induces an overall decrease of *Tfrc* mRNA hepatic level (HU: *P* < 0.001, Figure 2C), and only a trend toward TFRC protein levels decrease (HU: *P* = 0.073, Figure 2D,E). Regarding FPN expression, the response to HU is different between both sexes, with a decrease of the protein level in females, on contrary to males (−25.7% vs. −5.3%, Sex*HU: *P* = 0.022, Figure 1D,E) while in both HU groups, *Fpn* mRNA levels are unaffected (Figure 1C).

### Extreme physical inactivity differently affects spleen iron metabolism in female and male rats

Iron parameters and the expression of iron metabolism genes and proteins in the spleen of animals subjected to extreme physical inactivity are presented in Figure 2.

In control animals, similarly to that we found in the liver, spleen iron concentration (SIC) and H‐ferritin protein levels are higher in females compared with males (+76.1%, *P* < 0.001; +136%, *P* < 0.001, respectively Figure 2A,D,E). Concomitantly, control females exhibit lower levels of TFRC and FPN proteins compared with control males (−46.2%, *P* < 0.001 and −23.3%, *P* = 0.011, respectively, Figure 2D,E).

After 7 days of HU, both absolute and relative spleen weight did not differ in each sex compared with baseline (HU: *P* = 0.132 and *P* = 0.837, respectively). We observed an overall increase in SIC (HU: *P* = 0.003, Figure 2A), which is only significant in HU males compared with their controls (+30.1%, *P* = 0.023 Figure 2A). H‐ferritin protein level increase sharply in HU males compared with HU females (+60.9% vs. +1.14%, *P* = 0.007, Figure 2D,E). Perl's staining demonstrated that the iron excess was mainly localized in red pulp, which is a macrophage‐rich area (Figure 2B). Regarding TFRC expression, HU exposure induces a significant decrease of the protein level in males, contrary to females (−50.1% vs. −9.48%, Sex*HU: *P* = 0.003, Figure 2D,E) and an overall decrease in the mRNA level (HU: *P* = 0.004, Figure 2C), which is only significant in HU males (−47.3%, *P* = 0.017, Figure 2C).

Our data in spleen also shows an notable increase of heme oxygenase‐1 (HO‐1) and nuclear factor (erythroid‐derived 2)‐like 2 (NRF‐2) mRNA levels, these genes being involved in heme metabolism and defence against oxidative stress, in both HU males and females rats compared with their respective controls (HU: *P* < 0.001, Figure 2F). In males, contrary to females, these increases are associated with an increase of spleen *Fpn* mRNA level (+42.4% vs. −0.1%, Sex*HU: *P* = 0.031, respectively, Figure 2C) and an overall increase of spleen FPN protein levels (HU: *P* < 0.001), which is only significant in HU females compared with controls (+ 37.8%, *P* = 0.002, Figure 2D,E).

### Muscle adaptations of iron metabolism under extreme physical inactivity

Figure 3 presents the adaptations of iron metabolism in the soleus, a skeletal oxidative and rich‐iron muscle under extreme physical inactivity in male and female rats (Figure 3).

In control animals, we found that females have a higher muscle mass relative to body weight than males (49.7 ± 3.4 vs. 42.3 ± 5.7 mg/100 g, *P* = 0.0012). Soleus iron concentrations do not significantly differ between control males and females (Figure 3C).

After 7 days of HU, both males and females develop soleus atrophy characterized by a decrease in relative mass (−28.5%, *P* < 0.001 and −31.7%, *P* < 0.001; respectively) and a reduction in muscle fibre CSA (−45.3%, *P* < 0.001 and −38.9%, *P* < 0.001, respectively, Figure 3A,B). This muscle atrophy is associated with an overall increase in soleus iron concentrations (HU: *P* < 0.001) that is significant only in females compared with their controls (+26.7%, *P* = 0.004, Figure 3C), while only a trend is observed in males (+17.5%, *P* = 0.088, Figure 3C). These increases in HU males and females are associated with an overall rise of H‐ferritin protein levels (+748%, *P* < 0.001 and +369%, *P* < 0.001; respectively, Figure 3E,F) and a decrease of TFRC protein (−76.8%, *P* < 0.001 and −62.9%, *P* < 0.001, respectively, Figure 3E,F) and mRNA levels (−72.0%, *P* < 0.001 and −69.3%, *P* < 0.001, respectively, Figure 3D).

The myoglobin protein level increases regardless of sex after 7 days of extreme physical inactivity, despite the absence of an increase in myoglobin mRNA levels (HU: *P* < 0.001 and *P* = 0.494; respectively; Figure 3D–F). Similarly, cytochrome C protein levels increase in the soleus after 7 days of HU (HU: *P* = 0.006, Figure 3E,F), which is significant only in HU females (+20.0%, *P* = 0.029, Figure 3E). Concerning the expression of genes involved in Fe^2+^ and heme export, we found a significant decrease of FPN protein levels in both males and females (−68.9%, *P* < 0.001, and −75.9%, *P* < 0.001, respectively, Figure 3E,F). A significant increase of *Flvcr1* mRNA level was found in both males and females (+179.3%, *P* = 0.037 and +316.8%, *P* < 0.001, respectively, Figure 3D), whereas FLVCR1 protein levels in both sexes were decreased (−39.9% and −49.1%, respectively, HU: *P* < 0.001, Figure 3E,F).

## Discussion

In the present study, we investigated the impact of extreme physical inactivity on iron metabolism, and analysed the potential mechanisms involved in iron metabolism regulation in such condition in both males and females rats.

In Humans, in basal condition, women exhibit lower levels of ferritin, circulating hepcidin, and LIC compared with men—potentially due to menstrual bleeding and hormonal status.^23^, ^24^, ^25^ In rodents, we report here that female rats display on contrary higher levels of iron availability (serum iron and transferrin saturation), circulating hepcidin, LIC, and SIC than males, as previously reported.^26^, ^27^, ^28^ This difference in the relationship between iron parameters and sex between humans and rodents may be at least partly explained by the absence in animals of menstruation, which represents a subsequent source of iron loss in premenopausal women. Such elevated iron parameters could contribute to the very high reproductive rate observed in rats. Interestingly, most older women exposed to extreme physical inactivity, as patients confined to bed rest, do not experience menstrual bleeding, while astronauts often prefer to pharmacologically suppress menstruation during spaceflight for practical reasons.^29^, ^30^

In response to hindlimb unloading, male rats do not present an increase of circulating iron availability but exhibit an increase of serum hepcidin and SIC as reported in men.^4^, ^12^, ^13^ In addition, we also observed an LIC increase in rats, a finding not previously detected in men.^13^ We cannot exclude that the sensitivity level of MRI does not permit to identify a weak hepatic iron excess during extreme physical inactivity experiments, when values remain close to the normal ones. Importantly, the LIC increase we biochemically identified was not visible histologically using Perl's staining on liver slice, which is less sensitive than biochemical methods. Spleen and hepatic iron accumulation was associated to an increase H‐ferritin protein levels and a decrease in *Tfrc* mRNA and protein levels in both organs. Such gene expression adaptations are consistent with their potential regulation to cell iron content that could involve the IRE/IRP system at the post‐translational level, leading to an increase of H‐ferritin synthesis and a reduction in *Tfrc* mRNA levels. In the presence of iron excess, the IRE/IRP could theoretically also induce an increase of *Fpn* mRNA translation levels to promote cellular iron export.^31^ Interestingly, in male rats subjected to hindlimb unloading, there was no modulation of FPN protein levels both in spleen and liver. This lack of increase in FPN protein expression that we observed could be related to the increase of serum hepcidin levels, which may counteract translational overexpression of the protein by reducing or controlling its expression on the cell membrane.^8^

By contrast, female rats exposed to extreme physical inactivity did not exhibit an increase in transferrin saturation, serum iron, serum hepcidin levels, LIC or SIC. These data are consistent with our previous data obtained in bedridden women, which showed that iron availability and circulating hepcidin levels were not significantly modified in a small group (*n* = 7 women).^12^ All these data suggest that short‐term exposure to extreme physical inactivity induces an iron misdistribution in male, contrary to female rats. Such observations could be related to a higher capability of female rats to retain tissue iron. It is well known that, in both sexes, EPI favours an early anaemia, characterized by a decreased of haemoglobin mass.^4^, ^12^, ^32^ Although we do not observe significant haemoglobin level decrease in our study, a previous study conducted in rodents has shown that RBC count is reduced throughout exposition to tail suspension.^33^ Importantly, recent studies conducted in astronauts and bedridden volunteers have highlighted that EPI rapidly induce haemolytic processes (i.e., erythrophagocytosis and intravascular haemolysis).^2^, ^4^ Knowing that 70% of iron is associated to haemoglobin in red blood cells, the recycling of erythrocytes by macrophages, during erythrophagocytosis process could contribute to our observation in spleen and serum. Importantly, in both EPI groups, we found an increase of heme oxygenase‐1 (*HO‐1*) and nuclear factor (erythroid‐derived 2)‐like 2 (*NRF‐2*) mRNA levels in spleen, both genes being previously reported associated to erythrophagocytosis in spleen or in isolated macrophages (Delaby *et al*., 2012; Mathieu *et al*., 2014). We thus hypothesize that the iron excess in the spleen of male rats may result from both early erythrophagocytosis and the lack of increase of FPN protein expression in HU rodents. In females, the absence of spleen iron accumulation could be explained by the increase in spleen FPN protein probably favoured by the IRE/IRP system activation and the lack of an increase in serum hepcidin levels. Spleen iron egress may contribute to an increase of iron accumulation in others organs, such as bones, as observed in mouse exposed to 28 days of hindlimb unloading.^16^

Ten to fifteen per cent of body iron being present in skeletal muscle and involved in cellular respiration and energy metabolism,^21^, ^34^ we explored the responses of the soleus, an oxidative and iron‐rich muscle, that is early impacted by extreme physical inactivity. As reported in both humans and rodents,^35^ our data shows a drastic significant muscle atrophy in the soleus, characterized by reduced muscle mass and fibre CSA in both male and female rats. Concomitantly, animals of both sexes exhibited an increase in soleus iron concentration. The oxidative profile of the soleus requires a substantial amount of iron for energy production within the mitochondria. Iron is indeed complexed in iron–sulfur proteins (e.g., Cytochrome c) within the mitochondrial respiratory chain, as well as in myoglobin, a cytosolic metalloprotein responsible for oxygen storage.

Consistently with other experimental models promoting muscle atrophy or iron overload, muscle H‐ferritin protein concentrations increased in HU male and female rats, while TFRC protein levels decreased. This suggests once again an activation of the IRE/IRP system in skeletal muscle fibres.^36^, ^37^, ^38^ Interestingly, myoglobin protein levels are also increased in both HU groups, supporting the hypothesis of coordinated response between ferritin and myoglobin, as observed *in vitro* in rat L6G8C5 myotubes during iron exposure.^39^ Surprisingly, in both HU groups, the muscle levels of FPN protein were decreased. In males, this could be attributed again to the concomitant increase of serum hepcidin levels, but in female rats, as reported in liver and spleen, this decrease remains unexplained. Regarding the heme iron exporter *FLVCR1* gene, which may play a role in heme iron‐rich organ,^11^ mRNA levels increased while protein levels decreased in both HU groups, suggesting a potential post transcriptional regulation of FLVCR1. All together, these data suggest that a decrease of FPN and FLVCR1 protein expression could limit the export of intracellular free and heme iron during muscle atrophy. These results were consistent with the soleus iron concentration and myoglobin increases, suggesting that, under atrophy, iron seems to be not exported from muscle. Importantly, our previous data suggest that skeletal muscle can tolerate a high level of iron without major biological consequences.^21^ Further studies should explore muscle iron metabolism parameters during muscle atrophy after long‐term exposure to extreme physical inactivity.

**Figure 4:**
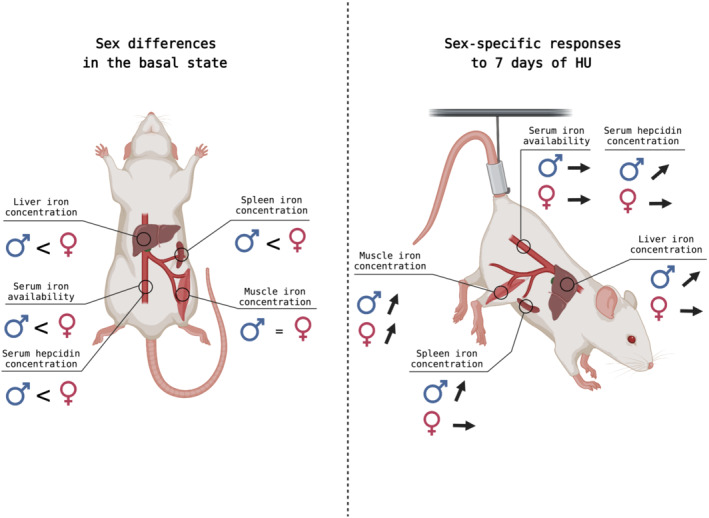
Graphical abstract illustrating the impact of extreme physical inactivity and sex on iron metabolism systemic regulation in rats. Unlike in humans, female rats exhibit higher serum, liver, and spleen iron concentrations and serum hepcidin levels than male rats in their basal state. However, there is no difference in iron concentration in the soleus muscle between the sexes in the basal state. In response to 7 days of hindlimb unloading, while both inactive males and females do not show altered plasma iron availability, only males present an increase in spleen and liver iron concentrations and serum hepcidin. However, both sexes exhibit an increase in iron concentration in the soleus muscle. This image was created with BioRender.com.

In summary, our data support that under extreme physical inactivity (1) contrary to females, male rats exhibit tissue iron accumulation and an increase in serum hepcidin levels as reported in human males (2) in both sexes, erythrophagocytosis could be a candidate for this iron misdistribution, whereas iron remains sequestrated in atrophied skeletal muscle (Figure 4).

## Conflict of interest

None.

## Funding

This study was supported by research grant from the French Centre National d'Etudes Spatiales (CNES ‐ grant number 480001119). M. Horeau received a PhD grant from CNES and Brittany Council.
